# A comprehensive dataset of eight Thai cannabis classes for botanical exploration

**DOI:** 10.1016/j.dib.2024.110292

**Published:** 2024-03-07

**Authors:** Kailas Patil, Prawit Chumchu

**Affiliations:** a Vishwakarma University, Pune, India; b Kasetsart University, Sriracha, Thailand

**Keywords:** Dataset, Cannabis plant, Leaf assessment, Plant health analysis

## Abstract

This dataset presents a comprehensive collection of images representing both dried and live samples from eight distinct Thai cannabis classes. The dataset includes a total of 14,094 images, with images depicting dried and healthy specimens. These images serve as a valuable resource for researchers engaged in botanical exploration, machine learning, and computer vision studies. Additionally, the dataset facilitates investigations into the medicinal properties of Thai cannabis. Interdisciplinary collaboration is encouraged, providing opportunities for innovative insights spanning biology, horticulture, and data science. Beyond fundamental research, this dataset holds practical implications for agriculture, technology development, and disease prevention, offering insights into both dried and live states of Thai cannabis plants across various strains.

Specifications Table

**Table:** 

Subject	Computer Science, Agricultural Science
Specific subject area	Agronomy & Crop Science, Computer vision, Image classification
Data format	Raw
Type of data	Image
Data collection	The data collection process encompassed capturing images of both dried and live samples from eight distinct Thai cannabis classes. A total of 14094 images were meticulously collected, comprising images of dried and healthy specimens. These images were saved in JPG format and underwent resizing to achieve a resolution of 1024 × 768 pixels using FastStone Photo Resizer. Special emphasis was placed on capturing images of dried plant leaves to provide comprehensive coverage of each cannabis class, including Foi Thong, Hang Kra Rog Phan ST1, Hang Suea Sakonnakhon TT1, KD KT, Kroeng Krawia, Purple Huai Khrai, Tanao Si Kan Khaw WA1, and Tanao Si Kan Dang RD1. This carefully executed data collection process resulted in a diverse and extensive dataset, offering valuable resources for researchers delving into image classification, machine learning, and computer vision within the realm of botanical studies.
Data source location	Kasetsart University Sriracha Campus, 199 Moo 6, Thungsukhla Subdistrict, Sriracha District, Chonburi Province 20230 Latitude: 12.785409° N Longitude: 101.024080° E
Data accessibility	(1) Repository name: Dataset of well-known Thai cannabis plants Data identification number: 10.17632/rd8c7fjrs8.2 Direct URL to data: https://data.mendeley.com/datasets/rd8c7fjrs8/2 (2) Repository name: Dataset of well-known Thai cannabis plantsData identification number: 10.17632/rd8c7fjrs8.3 Direct URL to data: https://zenodo.org/records/10635922

## 1 Value of the Data

• Researchers now have access to a comprehensive resource of 14094 images, encompassing both dried and healthy specimens, representing eight distinct Thai cannabis classes with images of both dried and healthy plant leaves.
• The diversity within the dataset presents a unique opportunity for scientists specializing in machine learning and computer vision to develop robust algorithms capable of precise image classification, encompassing both dried and healthy states of the plants.
• Ideal for studying medicinal properties, the dataset supports research into the therapeutic potential of different Thai cannabis classes, considering both their dried and healthy forms.
• Interdisciplinary researchers find opportunities for collaboration and innovative insights across biology, horticulture, and data science in this diverse dataset, while considering both the dried and healthy states of the plants.
• This dataset is a valuable resource with applications in research, agriculture, technology development, and disease prevention. It not only addresses essential questions but also provides practical tools for improving plant health assessment, ultimately contributing to more sustainable and efficient agricultural practices.

## 2 Data Description

The implementation of image processing and computer vision methods can serve as an alternative approach to accelerate the process of identifying or classifying plants. The dataset consists of a total of 14094 images, distributed across categories as shown in the Table 1.

**Table 1:** Distribution of Thai cannabis plant images.

The dataset comprises a collection of 14094 images, categorizing eight distinct Thai cannabis classes: Foi Thong, Hang Kra Rog Phan ST1, Hang Suea Sakonnakhon TT1, KD KT, Kroeng Krawia, Purple Huai Khrai, Tanao Si Kan Khaw WA1, and Tanao Si Kan Dang RD1. This diverse collection includes both dried and healthy specimens, offering researchers a comprehensive dataset for various research areas, including botanical exploration, machine learning, medicinal plant studies, education, and cross-disciplinary research initiatives.

Each category is organized within distinct folders, ensuring straightforward access and identification of specific samples. Refer to Fig. 2 for sample images of cannabis plant varieties included in the dataset.

## 3 Experimental Design, Materials and Methods

### 3.1 Experimental Design

The dataset images were captured using the Iphone 13 pro mobile phone, ensuring consistent image quality and resolution for each plant sample. Fig. 2 shows the experimental setup for dataset creation. The dataset encompasses eight categories, introducing variability in lighting and environmental factors to mimic real-world scenarios. Fig. 3 shows experimental configuration setup for the Thai Cannabis Plant dataset. Fig. 4 shows data acquisition process and Fig. 5 illustrates pre-processed image.

Step 1: Image Capture (September 2023) - In this step, we conducted field visits during both daytime and nighttime to capture images related to various conditions. The primary objective was to compile a comprehensive collection of images relevant to Thai Cannabis plant categories.

Step 2: Image Pre-processing (January 2024) - During this step, we enhanced the quality of Thai Cannabis plant images by resizing them to 1024 × 768 using FastStone Photo Resizer. The data acquisition process involved capturing images during field visits and subsequently preparing them through pre-processing for inclusion in the dataset.

### 3.2 Materials or Specification of Image Acquisition System

The mobile phone (Iphone 13 pro used in the data acquisition process and the specifications of the captured images are:

Sensor Type: 64 MP GW1 / S5KGW3
Focal Length: 26mm
Aperture Range: f/1.79-f/2.2
Aspect Ratio: 4:3

The images taken were saved in JPG format and were resized to a resolution of 1024 × 768 pixels using FastStone Photo Resizer. These specifications provide crucial details about the cameras used and the image properties obtained during the data acquisition process.

### 3.3 Preprocessing Method

In our study, we initiated image preprocessing using FastStone Photo Resizer, a versatile tool widely known for batch image resizing. This step, outlined in Fig. 6, streamlines the resizing process for image batches, proving valuable for preprocessing in diverse research applications, such as image-based machine learning, analysis, and data augmentation. For subsequent preprocessing, we adopted the 'preprocess_input' function within the Keras library, tailored for pre-trained models. This built-in function encompasses mean subtraction, channel reordering, scaling, and resizing, ensuring proper formatting of images for pre-trained models. Its role is crucial in guaranteeing that input images align with the specific requirements of the chosen pre-trained model.

Fig. 7 shows the augmentation code of Thai cannabis dataset and Fig. 8 represents image enhancement steps during preprocessing.

### 3.4 Demonstrating the Significance of the Thai Cannabis Plant Dataset

In the realm of machine learning datasets, several notable contributions have emerged recently [2], [3], [4], [5], [6], [7], [8],10] catering to machine learning applications. We wanted to show just how valuable our Thai Cannabis Plant Dataset [1,9] is, so we ran some experiments using well-known pre-trained models like VGG19, DenseNet201, and EfficientNetB7. Our goal was to see how this dataset can boost the accuracy of machine learning models, especially when it comes to identifying Thai Cannabis plants.

First, we ran these pre-trained models without any tweaks using our dataset as a sort of benchmark. Then, we gave these models a boost by training them on our dataset. What we found was pretty exciting. When we fine-tuned these models with our dataset, there was a significant jump in accuracy. This was most apparent in how well the models could detect and classify Thai cannabis plants. Table 2 shows accuracy of pretrained machine learning models on the Thai cannabis plant dataset before and after training with our dataset. Similarly, Table 3 shows confusion matrix of pretrained machine learning models on the Thai cannabis plant dataset before and after training with our dataset.

**Table 2:** Accuracy of pretrained machine learning models on the Thai cannabis plant dataset: before and after training.

Machine Learning Model	Accuracy (Before Training on our Dataset)	Accuracy (After Training on our Dataset)
VGG19	18.21%	99.67%
DenseNet201	9.92%	99.67%
EfficientNetB7	6.83%	99.67%

**Table 3:** Confusion Matrix of pretrained models.

**Fig. 1:**
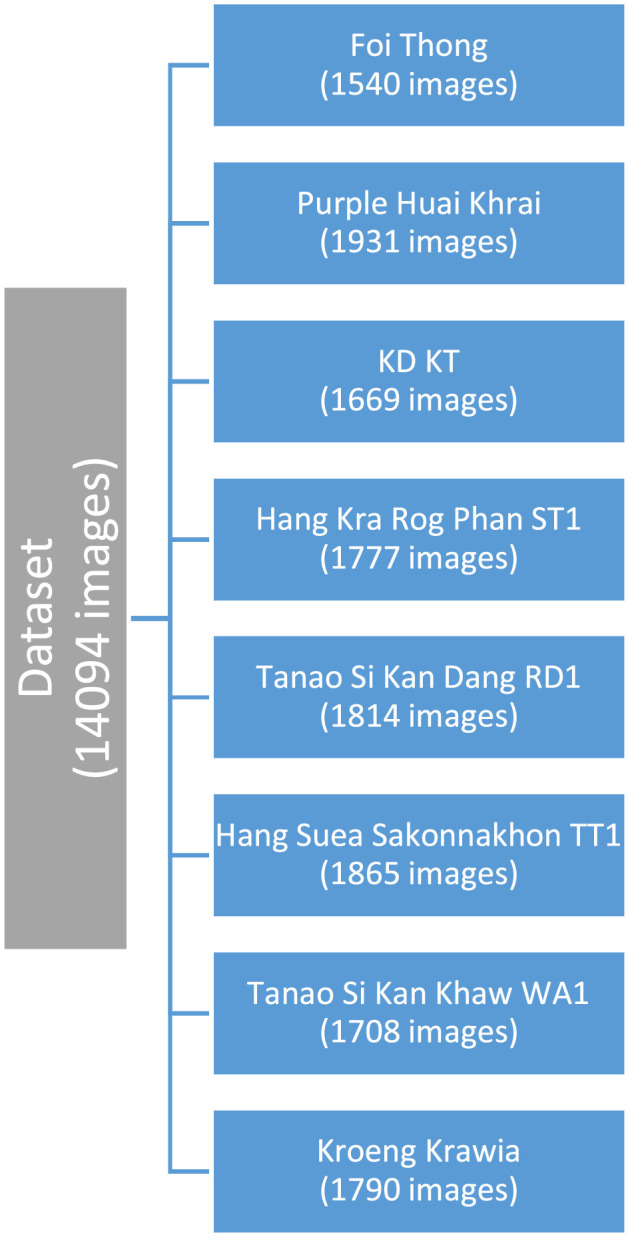
Organization of the cannabis plant dataset's folder structure.

In a nutshell, our dataset plays a crucial role in making these machine learning models, like VGG19, DenseNet201, and EfficientNetB7, perform much better. By offering a solid resource for training and fine-tuning, our dataset becomes a vital tool in creating more reliable models that can help improve Thai Cannabis Plant cultivation and keep those plants healthy (Fig. 1).

**Fig. 2:**
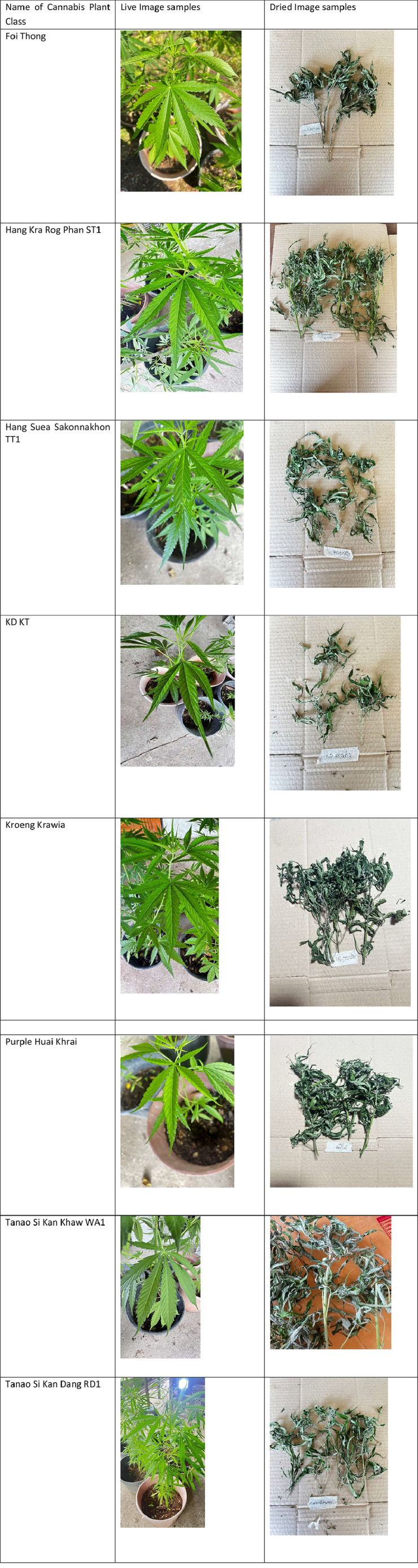
Sample images of cannabis plant varieties in the dataset.

**Fig. 3:**
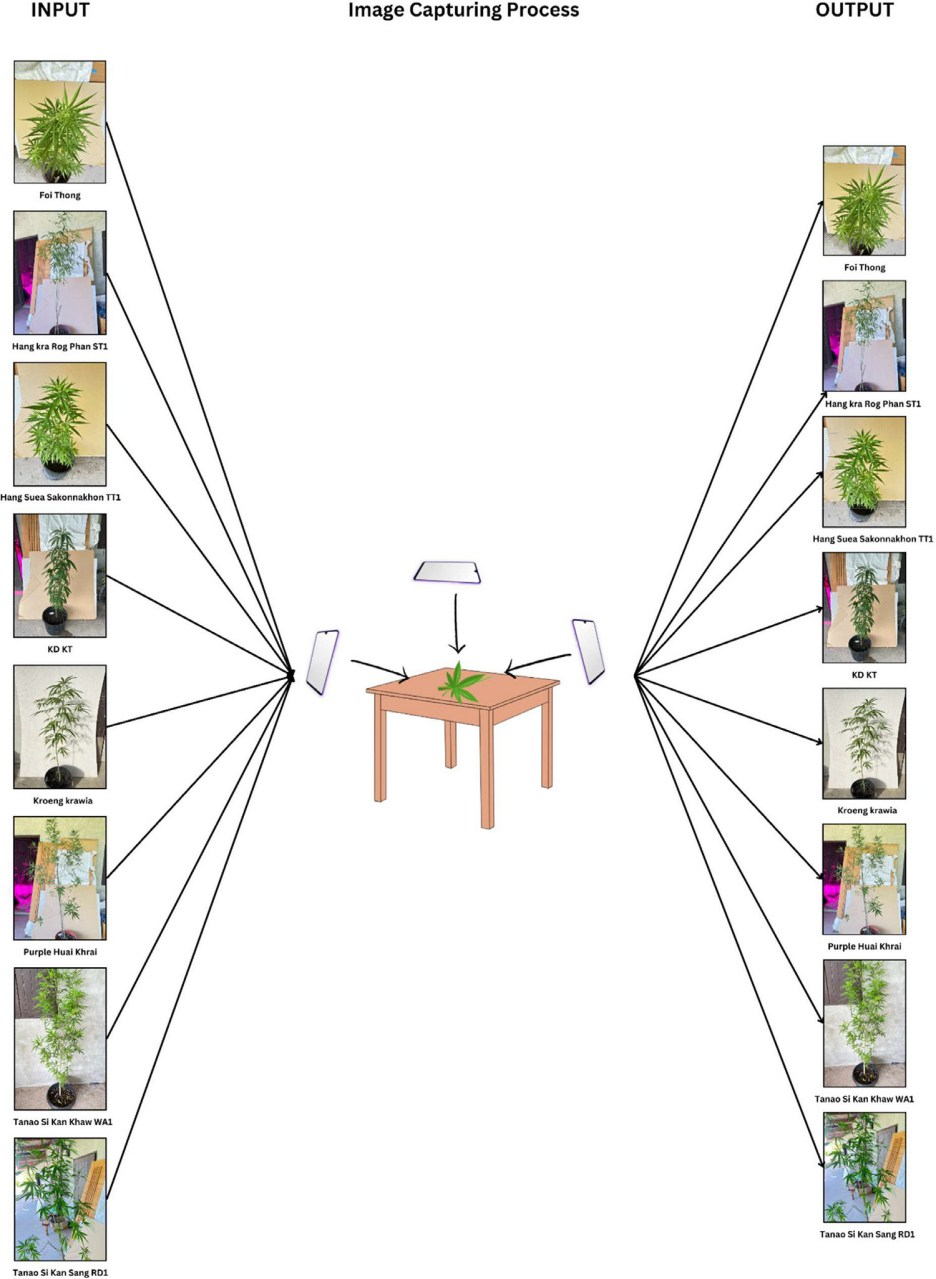
Experimental configuration for the cannabis plant dataset.

**Fig. 4:**
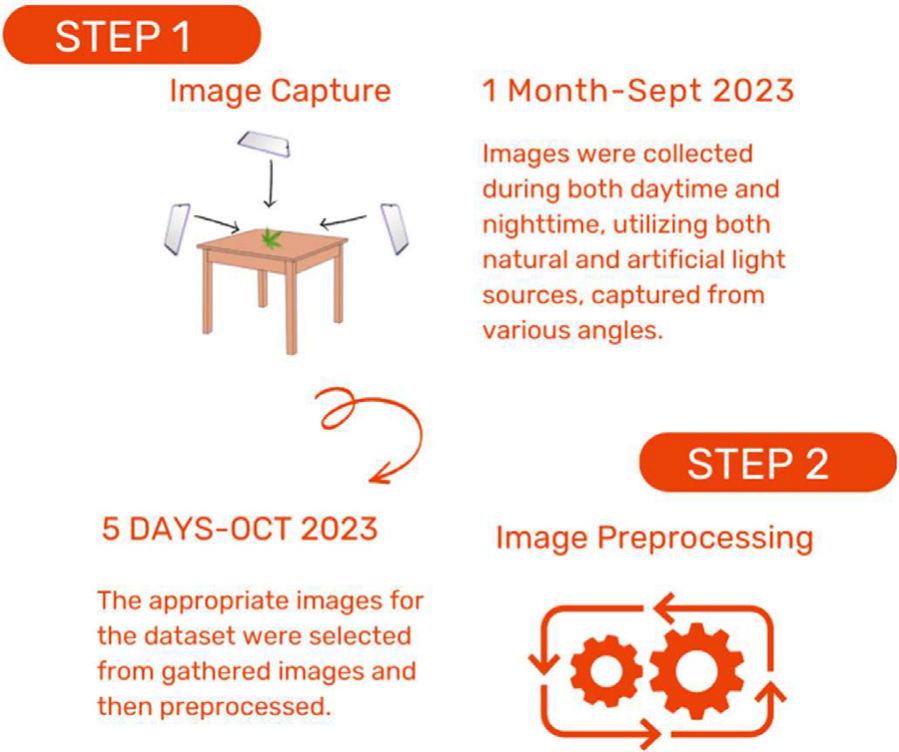
Data collection process.

**Fig. 5:**
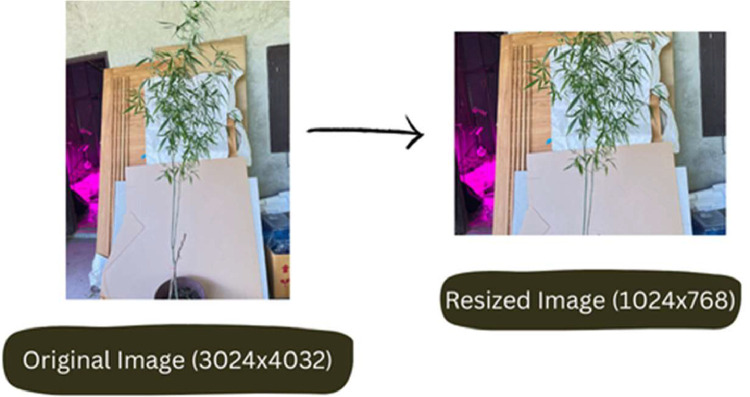
Pre-processed image.

**Fig. 6:**
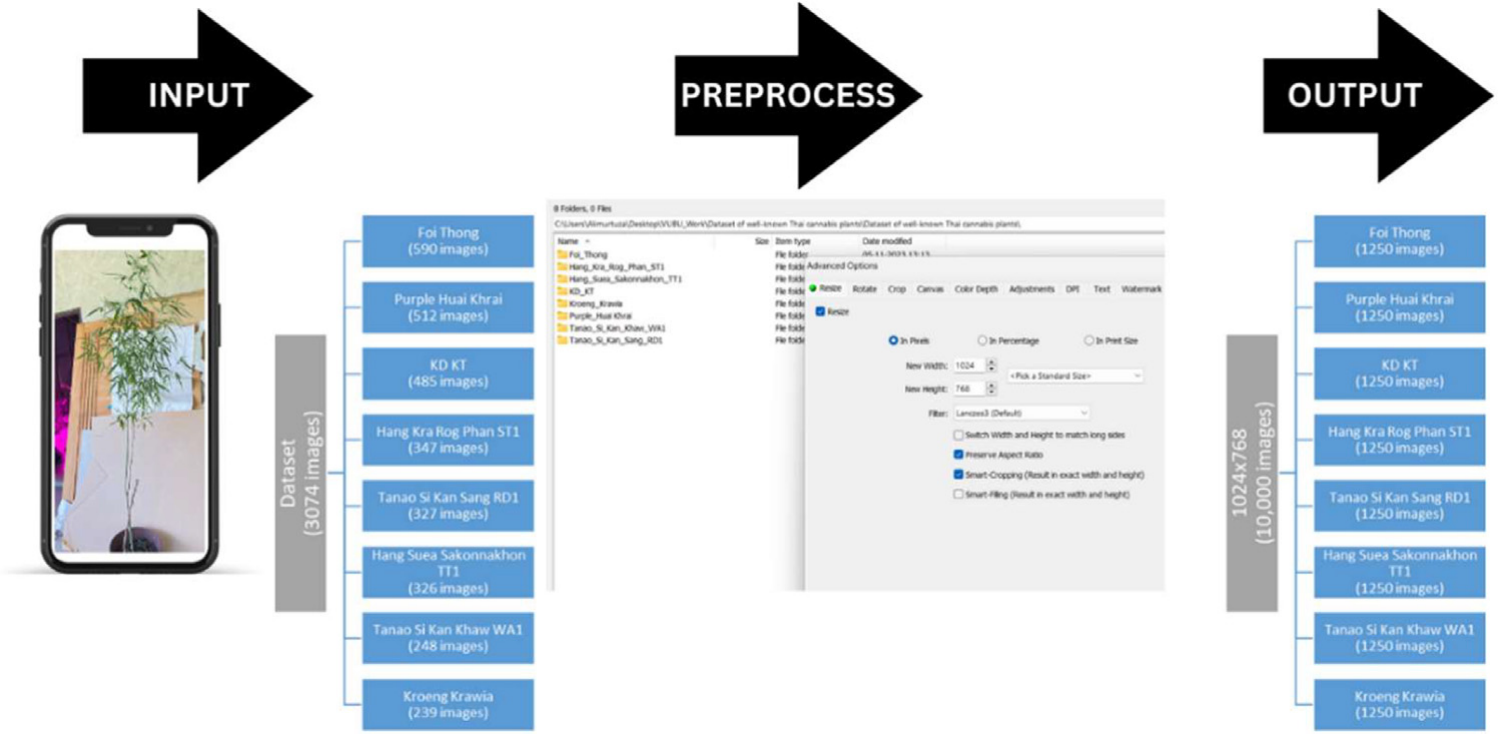
Stepwise preprocessing process.

**Fig. 7:**
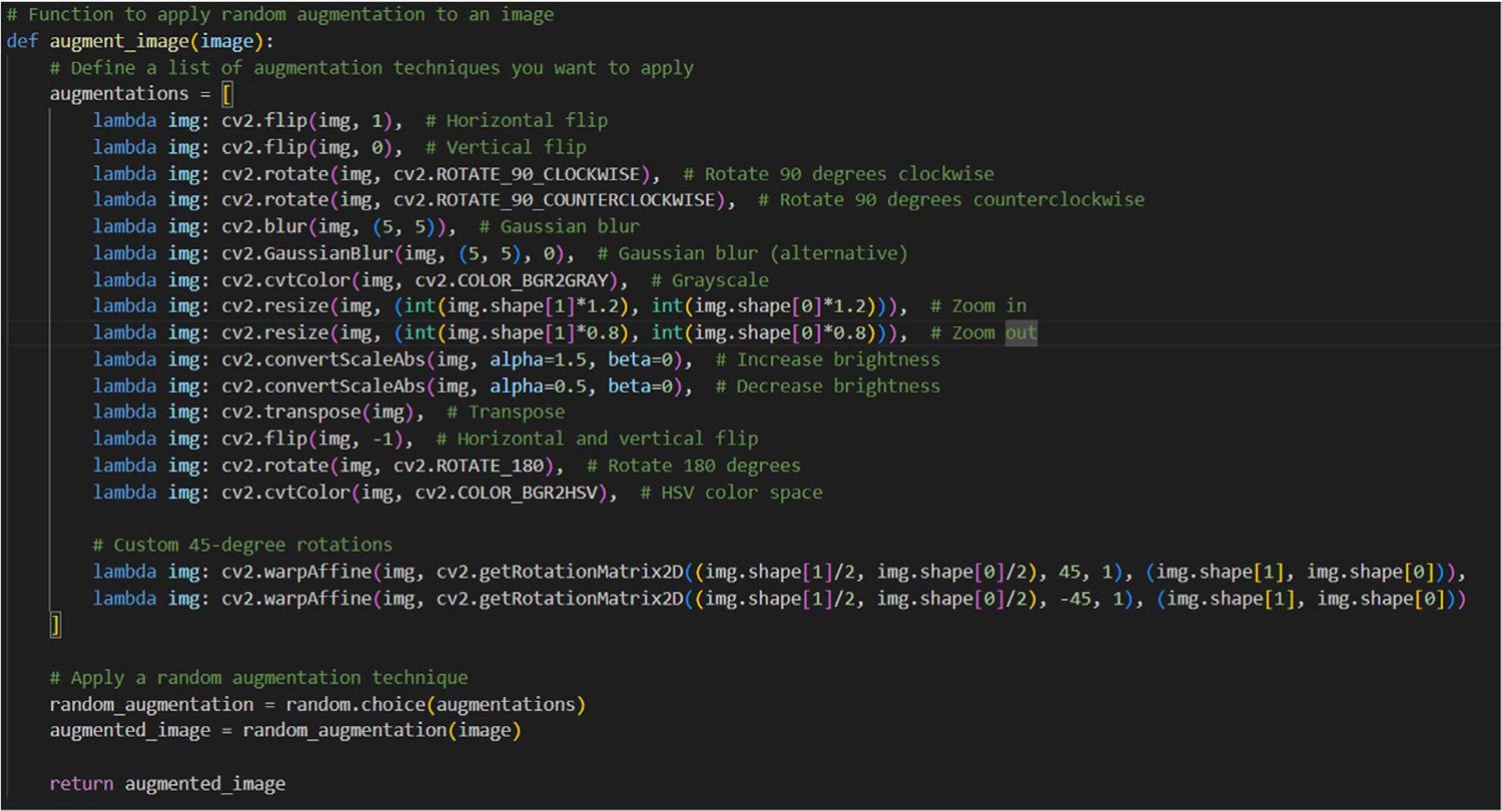
Augmentation code of Thai cannabis dataset.

**Fig. 8:**
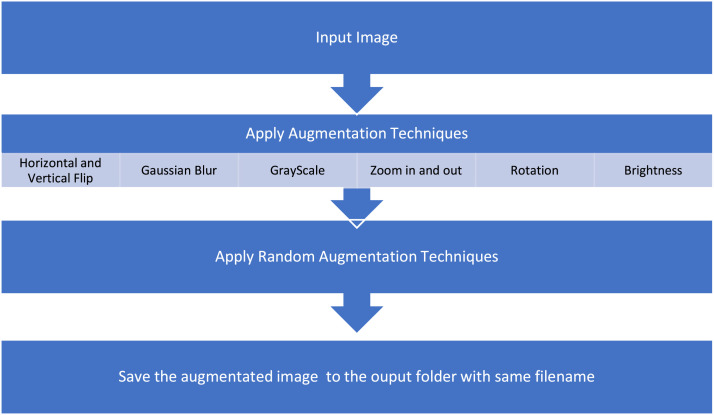
Flowchart of image enhancement steps.

The above confusion matrix in the Table 3, offers a detailed breakdown of the model's predictive accuracy. It allows us to discern where the model excels, correctly identifying instances within each class (true positives), and where it stumbles, making classification errors (predicted positives and predicted negatives).

## Limitations

Expanding the dataset to encompass a wider range of classes and samples from diverse global regions would enhance its overall diversity and applicability.

## Ethics Statement

Our study does not involve studies with animals or humans. Therefore, we confirm that our research strictly adheres to the guidelines for authors provided by Data in Brief terms of ethical considerations.

## CRediT authorship contribution statement

**Kailas Patil:** Conceptualization, Writing – review & editing. **Prawit Chumchu:** Methodology, Data curation, Supervision, Writing – review & editing.

## Data Availability

Dataset of well-known Thai cannabis plants (Original data) (zenedo). Dataset of well-known Thai cannabis plants (Original data) (Mendeley Data).
